# Supplementary material for: miR-483-5p offsets functional and behavioural effects of stress in male mice through synapse-targeted repression of Pgap2 in the basolateral amygdala
Source: Nat Commun. 2023 Apr 25;14:2134. doi: 10.1038/s41467-023-37688-2 (PMC10130081; doi:10.1038/s41467-023-37688-2)
Supplement: Supplementary file 1 — Supplementary information [file 41467_2023_37688_MOESM1_ESM.pdf]

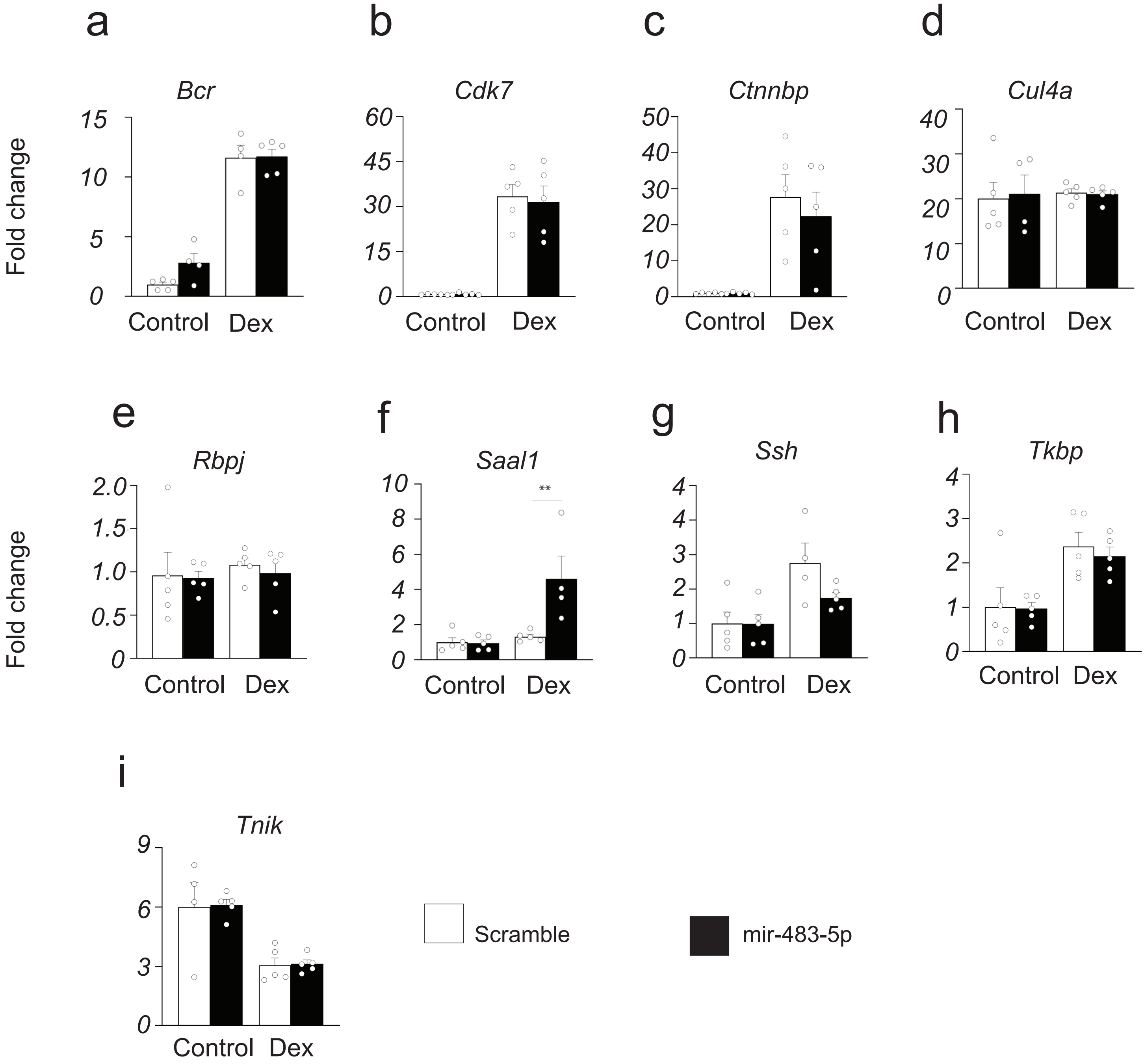

Supplementary Figure 1. miR-483-5p does not affect the expression of nine putative target genes.

Potential miR-483-5p targets were identified using the TargetScan algorithm and further analysed to detect genes expressed in the amygdala (cross-comparison with the ElMMo database) and regulated in response to stress (filtered through the AmiGo search engine). Upon identification of the genes of interest, Neuro2a cells were transfected with the miR-483-5p-expressing vector (or a scrambled control vector), treated with dexamethasone (or vehicle), and gene expression levels of *Bcr*, *Cdk7*, *Ctnnbp*, *Cul4a*, *Rbpj*, *Saal1*, *Tkbp*, *Tnik,* and *Shh* measured by quantitative RT-PCR. Data are presented as dot plots with mean±SEM; n = 5 animals per group; statistical significance is assessed by 2-way ANOVA with Šídák's multiple comparisons test as a post hoc test, **p<0.01 vs an indicated group. Source data and the exact p-values are provided in a Source Data file.

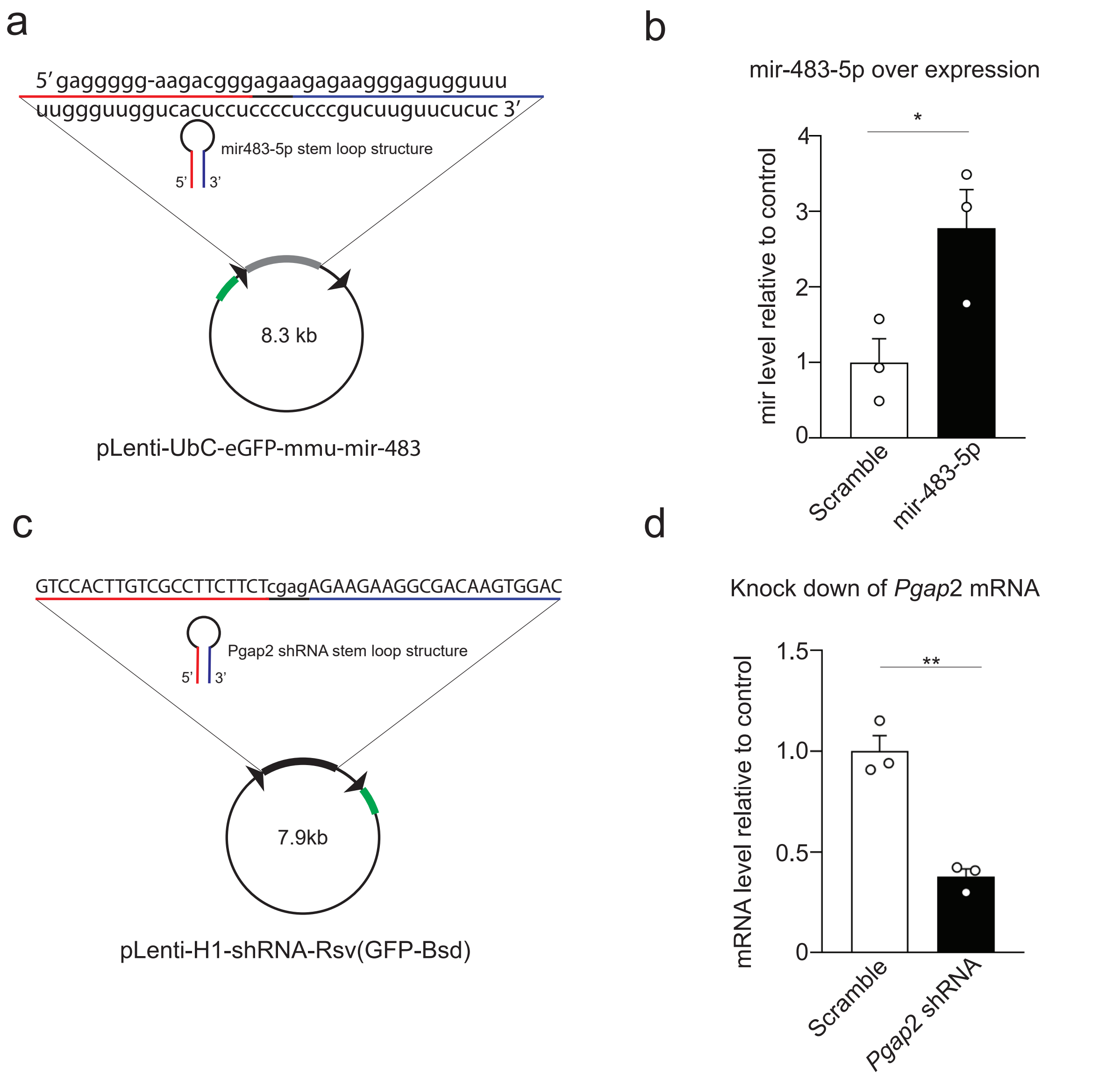

Supplementary Figure 2. The design, composition and efficiency of the lentiviral transduction vectors.

(a) miR-483-5p sequences (or a control scramble sequences) were inserted into the pUltra plasmid and lentiviral particles produced in HEK293t cells (pLenti-UbC-eGFP-mmu-mir-483). The miR-483-5p insert was positioned downstream of an enhanced green fluorescence protein (EGFP). (b) Transduction of Neuro2a cells with the pLenti-UbC-eGFP-mmu-mir-483 produced 3-fold increase in miR-483-5p expression as examined by quantitative RT-PCR. (c) To knock-down *Pgap2,* shRNA stem loop structure (or scrambled control sequence) was inserted into the lentiviral vector driving the expression through the H1 promoter (pLenti-H1-shRNA-Rsv(GFP-Bsd)). (d) Transduction of HEK293T cells with the pLenti-H1-shRNA-Rsv(GFP-Bsd) resulted in ~60% decrease in *Pgap2* expression as examined by quantitative RT-PCR. Data are presented as dot plots with mean±SEM; n = 3 animals per group; statistical significance is assessed by unpaired two-sided t-test, *p<0.05, **p<0.01 vs an indicated group. Source data and the exact p-values are provided in a Source Data file.

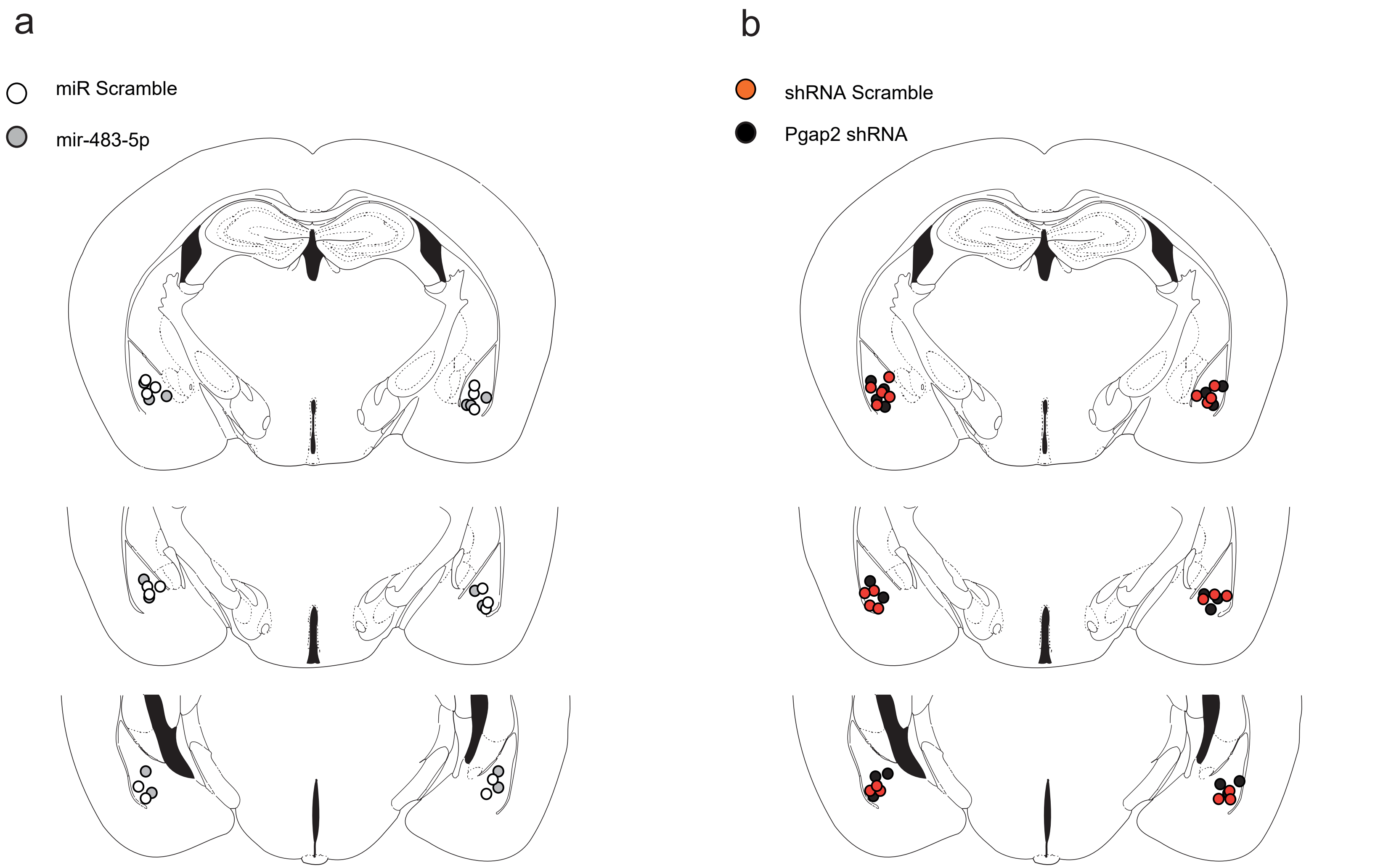

Supplementary Figure 3. Histological verification of stereotaxic amygdala injections.

After the completion of behavioural experiments mouse brains were harvested and immunostained to visualize the green fluorescent protein reporter (GFP). The positions of the injection needle tips for the miR-483-5p overexpression (a) and *Pgap2* knock-down experiments (b) are shown on consecutive coronal brain atlas planes.

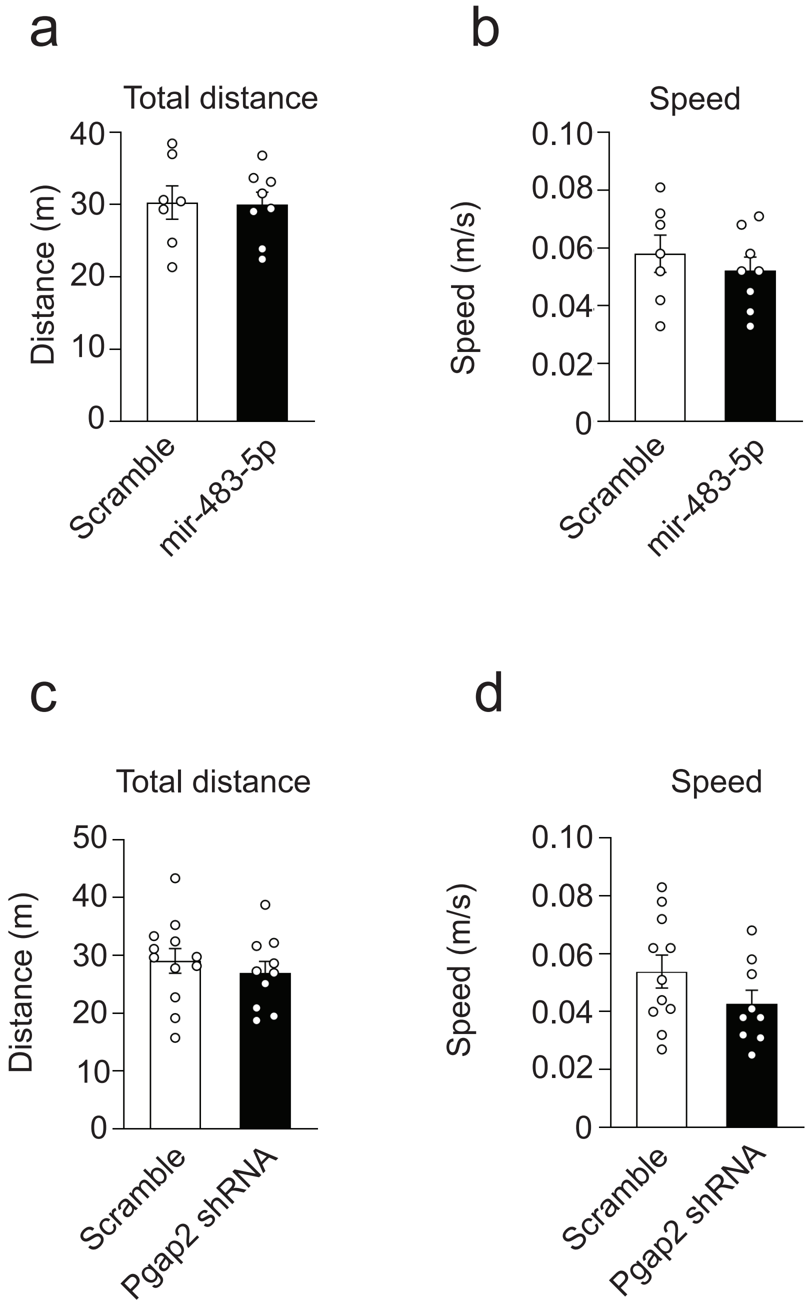

Supplementary Figure 4. Lentiviral gene transfer in the basolateral amygdala does not affect locomotor activity of mice.

Lentivirus-mediated overexpression of miR-483-5p in the basolateral amygdala did not affect the total distance travelled (a) or average speed (b) of mice in the elevated-plus maze, as compared with mice injected with the vector containing control scrambled sequence. Similarly, shRNA-mediated suppression of *Pgap2* in the basolateral amygdala did not affect the total distance travelled (c) or average speed (d) of mice in the apparatus in comparison to mice injected with the vector containing control shRNA sequence. Data are presented as dot plots with mean±SEM; n = 7-12 animals per group; statistical significance is assessed by unpaired two-sided t-test. Source data and the exact p-values are provided in a Source Data file.

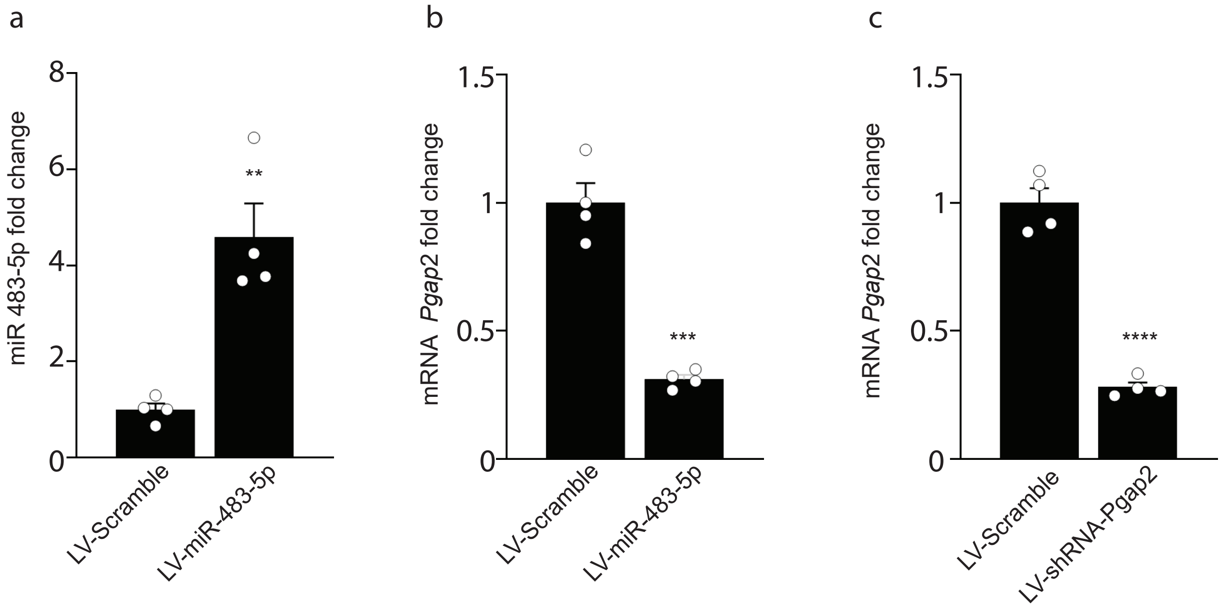

Supplementary Figure 5. Stereotaxic injection of the lentivirus overexpressing miR-483-5p an shRNA targeting *Pgap2* mRNA leads to downregulation of the *Pgap2* gene.

Injection of the miR-483-5p expressing lentiviral particles in mouse basolateral amygdala provides the stable expression of the miR molecules (a) and triggers significant reduction of the *Pgap2* coding mRNA levels (b). Similarly, expression of the *Pgap2* targeting shRNA leads to decrease of the *Pgap2* coding mRNA levels (c). Data are presented as dot plots with mean±SEM; n = 4 animals per group; statistical significance is assessed by unpaired two-sided t-test, ****p<0.0001, ***p<0.001, **p<0.01 vs scramble. Source data and the exact p-values are provided in a Source Data file.
